# Achievements and memories of Dr. Takashi Sugimura

**DOI:** 10.1186/s41021-021-00203-4

**Published:** 2021-07-20

**Authors:** Keiji Wakabayashi

**Affiliations:** 1 National Cancer Center Research Institute, 1-1, Tsukiji 5-Chome, Chuo-ku 104-0045 Tokyo, Japan; 2 Graduate Division of Nutritional and Environmental Sciences, University of Shizuoka, 52-1 Yada Suruga-ku, Shizuoka, 422-8526 Japan

In 1915, Dr. Katsusaburo Yamagiwa applied coal tar to the ears of rabbits and succeeded in inducing cancer in laboratory animals for the first time in the world, laying the foundation for the history of chemical carcinogenesis in Japan. This was followed by liver carcinogenesis in rats by Dr. Tomizo Yoshida and skin carcinogenesis in mice by Dr. Waro Nakahara. In 1966–67, Dr. Takashi Sugimura injected MNNG, a mutagen, subcutaneously into rats to induce sarcoma, and also induced gastric cancer in rats by giving drinking water containing MNNG. These achievements were pioneering in showing the relationship between mutagens and carcinogens, suggesting that carcinogenesis is caused by genetic changes, and have been highly evaluated internationally. For these achievements, Dr. Sugimura was awarded the Order of Culture.

With this history as a background, Dr. Sugimura of the National Cancer Center Research Institute (NCCRI), Dr. Yataro Tajima and Dr. Tsuneo Kada of the National Institute of Genetics, Dr. Motoi Ishidate Jr. of the National Institute of Hygienic Sciences (Now National Institute of Health Sciences), Dr. Sohei Kondo of Osaka University, and others took the lead in founding the Japanese Environmental Mutagen Society (JEMS) in 1972. The following year (1973), the mutagenicity of AF-2, a nitrofuran derivative used as a food preservative, was reported by Drs. Kondo and Kada, and its use was subsequently banned due to its proven carcinogenicity. I heard that JEMS, which was established under such circumstances, had a lot of momentum, and all the members of the society boldly took on the many difficult scientific problems that lay before them. Dr. Sugimura served as the president of the 5th Annual meeting of JEMS in Tokyo in 1976, and his strong leadership contributed to the great progress of early environmental mutagen research in Japan. In 1981, the 3rd International Conference on Environmental Mutagens was held in Tokyo, contributing to the improvement of international exchange in environmental mutagen research. As a result of these activities, many outstanding achievements originating from this society were made during the 1970s and 1980s, and the international status of JEMS was established. Some of them are described below.

1. Proof of mutagenicity of AF-2 (Sohei Kondo & Tsuneo Kada group)
2. Discovery of mutagenic and carcinogenic heterocyclic amines (HCAs) in heated foods (Takashi Sugimura & Minako Nagao Group).
3. Development of a method for detecting carcinogens using chromosomal aberrations as an indicator (Motoi Ishidate Jr. & Toshio Sofuni Group)
4. Metabolic activation mechanism of environmental mutagens and carcinogens (Ryuichi Kato & Tetsuya Kamataki Group)
5. Identification of mutagenic pollutants in the atmosphere (Hiroshi Tokiwa & Yoshinari Ohnishi Group)
6. Development of Blue cotton and Blue rayon (Hikoya Hayatsu Group)
7. Identification of 8-OH-dG caused by oxidative damage to DNA (Susumu Nishimura & Hiroshi Kasai Group)

I feel that times are changing since Dr. Sugimura, Dr. Tajima, Dr. Kada, Dr. Ishidate, and Dr. Kondo, who contributed to the establishment of this society, have passed away.

The first time I met Dr. Sugimura was about 45 years ago, when he was Director of NCCRI. I heard that he gazed at smoke rising from the grilling of fish in his kitchen and thought, “This smoke must include carcinogens because we now know there are carcinogens in cigarette smoke”. He and Dr. Nagao immediately bought fish from the neighboring Tsukiji market, and collected the smoke from the grilled fish and examined its mutagenicity using *Salmonella typhimurium*. Strong mutagenicity was observed in the TA98 strain in the presence of S9 mix. The mutagenicity was found not only in the smoke, but also in the charred flesh of the fish. Similarly, mutagenic activity was observed in grilled beef and hamburgers. It was also found that heating proteins and amino acids produced strong mutagenic activity. At that time, I was a doctoral student at Shizuoka College of Pharmacy (later, merged into the University of Shizuoka), and studying substances that showed antihistamine and antibacterial activity in tar obtained by heating proteins and amino acids at high temperatures, under the supervision of Professor Takuo Kosuge. One day, Dr. Takashi Kawachi (Deputy Director of NCCRI at that time) visited the university, and joint research began between NCCRI, Shizuoka College of Pharmacy and Faculty of Pharmaceutical Science, University of Tokyo. We have been searching for mutagens contained in cooked foods such as grilled fish and meat. A series of more than 20 HCAs, including Trp-P-1 and Trp-P-2, have been identified as mutagens. This was a good time when we could pursue the essence of science without worrying about the impact factor or the number of citations of a paper.

This joint research led me to obtain a position as on the research staff at NCCRI in 1979. Subsequently, 10 HCAs identified as mutagens were shown to be carcinogenic in mice and rats. Currently, a wide variety of studies on HCAs, including metabolic activation mechanisms, DNA-adduct structures, mutagenic expression mechanisms, human exposure doses, and epidemiological studies, have been reported by many research institutes around the world. These have been highly evaluated both domestically and internationally as extremely useful concrete examples of the association between dietary factors and human carcinogenesis.

I worked at NCCRI for 30 years. During that time, under the supervision of Dr. Sugimura, I conducted research on “HCAs as mutagens and carcinogens in heated foods,” “Research on the mechanism of co-mutagenic action of β-carboline compounds,” “Research on novel nitroso compounds in foods,” “Identification of novel mutagenic compounds in river water”, “Research on cancer chemoprevention of colorectal cancer”, and “Research on ADP-ribosylation of DNA”. Through these studies, I believe that we have made some achievement and contribution to the advancement in research fields on carcinogenesis and cancer prevention. In the meantime, Dr. Sugimura gave us the following advice: “Seek a significant difference of five to ten times or more! Within a factor of two times is the margin of error,” “Grasp the big flow of research and pursue the truth,” and “Improve your research intuition and catch a glimpse of what you are pursuing quickly.” His words taught me the importance of getting to the heart of the research.

I have accompanied Dr. Sugimura to many domestic and overseas academic conferences, and many memories come back to me as if it were yesterday. I cannot thank him enough for his kindness and support.

## Abbreviations

MNNG: *N*-Methyl-*N′*-nitro-*N*-nitrosoguanidine
AF-2: 2-(2-Furyl)-3-(5-nitro-2-furyl)acrylamide
8-OH-dG: 8-Hydroxy-2′-deoxyguanosine
Trp-P-1: 3-Amino-1,4-dimethyl-5*H*-pyrido [4, 3-*b*]indole
Trp-P-2: 3-Amino-1-methyl-5*H*-pyrido [4, 3-*b*]indole

